# From Help-Seekers to Influential Users: A Systematic Review of Participation Styles in Online Health Communities

**DOI:** 10.2196/jmir.4705

**Published:** 2015-12-01

**Authors:** Bradley Carron-Arthur, Kathina Ali, John Alastair Cunningham, Kathleen Margaret Griffiths

**Affiliations:** ^1^ National Institute for Mental Health Research Research School of Population Health The Australian National University Acton Australia; ^2^ Centre for Addiction and Mental Health Toronto, ON Canada

**Keywords:** online health community, participation style, social network, participation inequality, systematic review

## Abstract

**Background:**

Understanding how people participate in and contribute to online health communities (OHCs) is useful knowledge in multiple domains. It is helpful for community managers in developing strategies for building community, for organizations in disseminating information about health interventions, and for researchers in understanding the social dynamics of peer support.

**Objective:**

We sought to determine if any patterns were apparent in the nature of user participation across online health communities.

**Methods:**

The current study involved a systematic review of all studies that have investigated the nature of participation in an online health community and have provided a quantifiable method for categorizing a person based on their participation style. A systematic search yielded 20 papers.

**Results:**

Participatory styles were classified as either multidimensional (based on multiple metrics) or unidimensional (based on one metric). With respect to the multidimensional category, a total of 41 different participation styles were identified ranging from Influential Users who were leaders on the board to Topic-Focused Responders who focused on a specific topic and tended to respond to rather than initiate posts. However, there was little overlap in participation styles identified both across OHCs for different health conditions and within OHCs for specific health conditions. Five of the 41 styles emerged in more than one study (Hubs, Authorities, Facilitators, Prime Givers, and Discussants), but the remainder were reported in only one study. The focus of the unidimensional studies was on level of engagement and particularly on high-engaged users. Eight different metrics were used to evaluate level of engagement with the greatest focus on frequency of posts.

**Conclusions:**

With the exception of high-engaged users based on high post frequency, the current review found little evidence for consistent participatory styles across different health communities. However, this area of research is in its infancy, with most of the studies included in the review being published in the last 2 years. Nevertheless, the review delivers a nomenclature for OHC participation styles and metrics and discusses important methodological issues that will provide a basis for future comparative research in the area. Further studies are required to systematically investigate a range of participatory styles, to investigate their association with different types of online health communities and to determine the contribution of different participatory styles within and across online health communities.

## Introduction

Participation rates of people in online communities are known to be highly variable with some people contributing much more than others. Across all types of online communities, the variability in degree of user participation consistently follows a pattern [1]. In particular, this pattern in participation is described by a power law. This power law means, for example, that the top 1% of participants contribute as much as 75% of the posts in an online health community (OHC) [2,3]. This pattern is indicative of a coherent community [2], and these highly engaged individuals are repeatedly observed in well-established OHCs [4]. These individuals are of interest. Their high participation rates and predictable presence suggest that they may be of particular value to the OHC.

Although post frequency may constitute a simple indicator of engagement, from post frequency alone it is not possible to ascertain exactly what ways a person contributes. Post frequency does not indicate whether a person starts new discussions, welcomes newcomers, is available at critical times in the day when people are most likely to need support, or is knowledgeable about certain topics. In order to ascertain whether people contribute these different kinds of value, it is necessary to measure their participation based on various other metrics.

There may be value for those who are involved in the development of an OHC to identify users who contribute particular types of value to the OHC. This points to the need for multiple metrics to define user contributions. For example, in a qualitative paper on building and sustaining OHCs, Young described how certain core members were vital to the development and sustainability of an OHC [5]. As the community manager from the inception of this OHC, Young was able to provide an account of the different ways that these users had contributed to the development of the OHC including facilitating discussion and fostering a supportive culture. Young also suggested ways that OHC managers might harness the contributions of these individuals to help build the community by, for example, highlighting their best posts or inviting them to contribute to a community resource such as a newsletter.

For a variety of reasons, including time constraints and size of the community, not all community managers are able to have a strong qualitative understanding of the roles of particular individuals in their OHC. However, community managers would potentially benefit from a simple operationalization of user participation in terms of metrics that are automatically collected in the log data of the OHC software. This would help them to identify the core members and various other users who contribute in different ways so that they may apply the community building techniques recommended by Young [5].

OHCs also provide an opportune setting for interventions that encourage certain positive health behaviors [6]. Knowing who the most influential people are in an OHC, or how to reach most of the community via the smallest subset of people, might inform dissemination activities such as promoting new evidence-based treatments or recommending correct use of certain medications.

Finally, there is scientific value in investigating the ways in which different people participate in OHCs across multiple contexts. There may be patterns in the way in which people participate that can be found across multiple different OHCs. These patterns may help us learn more about the social dynamics of OHCs and the way that people seek help and provide it to others.

User profiling by categorizing participation styles is conducted in studies of online communities more broadly. There are some roles such as “newbies” and “celebrities” that may be found in any online community, but most others are likely to be specific to the type of community [7]. For example, “technical editors” and “substantive experts” are found in Wikipedia [8], but these may not be relevant to or found in OHCs. We expect that OHCs will have high-profile users who are akin to “celebrities,” but the nomenclature and the metrics used to define these users may be tailored to the supportive context and health discussion focus of the community. There may be further similarities and differences between participation styles in communities of different health types.

This study seeks to advance this area by conducting a systematic review of all studies that provide replicable, quantifiable criteria for categorizing the nature of participation in an OHC. We aimed to document all participation styles that had been identified to date and the OHCs from which they came. Our objective was to determine if any patterns were apparent in the nature of user participation across OHCs for different health conditions or within each.

## Methods

A systematic review was conducted to identify articles that investigated participation styles in an online health community. For the current purposes, an online health community was defined as any Internet-based platform designed to enable people to communicate about health issues. A participation style was defined as any type of engagement with an OHC that can be measured quantitatively. This does not include simply the presence or absence of participation (ie, posters and lurkers), as this has been well documented elsewhere [9], but rather is aimed at understanding the nature of participation for those who are actively engaged in the community.

### Search Strategy

Three databases (PubMed, PsycINFO, and Cochrane) were searched for all articles prior to December 2014. Adapted search terms from Eysenbach et al [10] and Griffiths et al [11] were used to identify the concept of OHC (see Multimedia Appendix 1). These search terms were combined with the following terms to identify the participation style concept: (participatory pattern*) OR (posting pattern*) OR (posting behavior pattern*) OR (use pattern*) OR (communication pattern*) OR (usage pattern*) OR (system use*) OR (traffic) OR (participative stance*) OR (participant contribution*) OR (posting habits*) OR (participation rate*) OR (posting rate*) OR (user engagement) OR (level* of engagement*) OR (pattern* of engagement*) OR (type* of engagement) OR (share information) OR (community structure) OR (social dynamics).

In addition, papers from relevant journals and conference proceedings in the computer and information science field published since 2005 (including the *American Medical Informatics Association Annual Symposium*, *Journal of the American Medical Informatics Association*, *Journal of the Association for Information Science and Technology*, and *International Conference on Healthcare Informatics*) and a new journal that was not yet indexed at the time of the search (*Internet Interventions*) were screened for relevant articles.

### Article Selection

**Figure 1:**
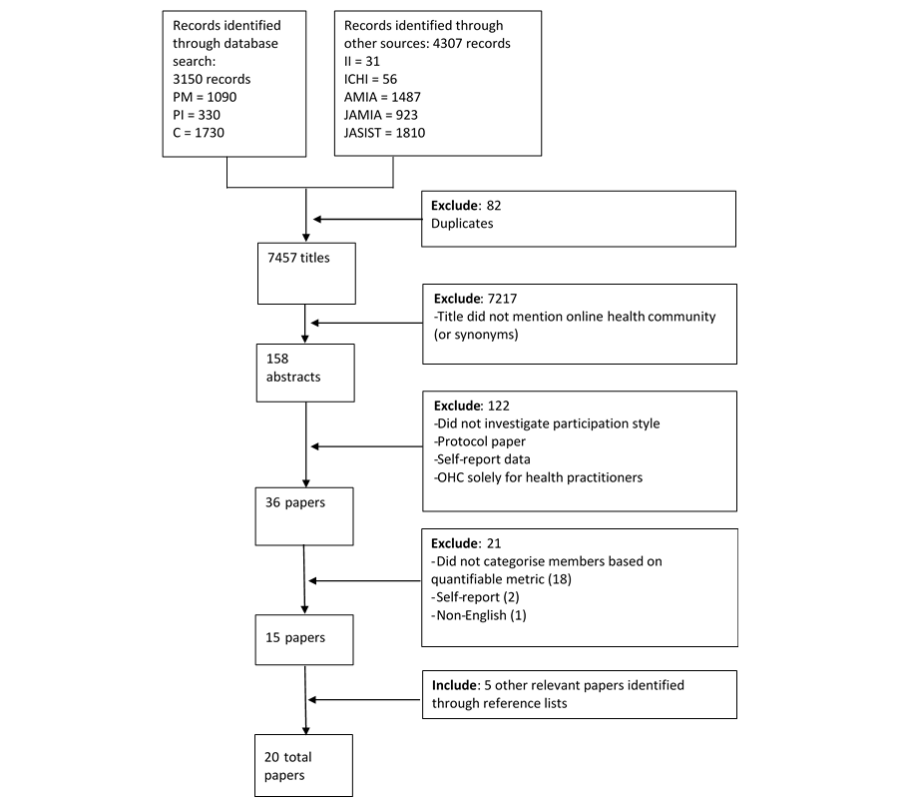
Study identification flow diagram: PubMed (PM), PsychINFO (PI), Cochrane (C), Internet Interventions (II), International Conference on Healthcare Informatics (ICHI), American Medical Informatics Association Annual Symposium (AMIA), Journal of the American Medical Informatics Association (JAMIA), Journal of the Association for Information Science and Technology (JASIST).

A total of 7457 articles were screened. Of these, 3150 were retrieved from the database search and 4307 were from the additional journals and conference proceedings. A total of 82 duplicate articles were identified and removed. Relevant articles were selected through a multistage process (Figure 1). Initially, titles were screened by 2 raters (BC and KA). Any article that mentioned an online community or synonym thereof in the title (or online health community in the case of the *Journal of the Association for Information Science and Technology*) was included. This reduced the number of articles to 158. The abstracts of these articles were subsequently screened by the 2 raters. Any article that investigated ways that people participate in an online health community was included. Articles based on self-report measures of OHC use and research protocols were excluded. The full articles for the 36 remaining abstracts were retrieved and read by both raters. Any disagreements between the raters were resolved by discussion.

#### Inclusion Criteria

The final set of articles included any study that (1) quantitatively investigated ways that people participate in an online health community, and (2) categorized users based on any quantifiable metric that can be used to show they have engaged with the community.

#### Exclusion Criteria

Studies that converted written content to quantitative data by a means that was computerized (eg, machine learning algorithm) were included, but studies that relied on human interpretation of written content to create quantitative data were not. This ensured that the methods identified could be accurately replicated and would be scalable to large OHCs. For similar reasons, studies that used self-report data from surveys were not included. This meant that only studies reporting data that had been automatically logged by the OHC software or that had been extracted by programs that crawl publicly available data were included in this systematic review. Protocol papers, articles not written in English, and papers on OHCs solely for health practitioners were not included.

After applying these criteria, a set of 15 papers were included. The reference lists of included papers and those that cited them (as per Google Scholar) were hand searched. This yielded an additional 5 papers, resulting in a final set of 20 included papers.

### Coding

The included papers were coded by 1 rater (BC). Each participation style identified by a paper was listed. Three attributes of each participation style were coded: (1) the name used by the authors to describe the participation style, for example, “superuser,” (2) the metrics used to quantitatively describe their style of participation, for example, frequency of posts, and (3) the inclusion criteria used to determine who was categorized as having that participation style, for example, the top 1% of users whose frequency of posts was greatest were deemed to be superusers.

## Results

Across the final set of 20 papers, users were categorized into participation styles a total of 74 times, of which 28 were duplicates. These duplicates included participation styles that had been assigned different names by different studies but used the same metrics and same inclusion criteria (or very similar) to define them. By merging all these redundant categorizations into the same participation style, we determined that 44 participation styles had been identified in OHCs to date.

Table 1 [2,3,12-29] shows a summary of information about the OHCs where the participation styles were identified. Some studies investigated more than one OHC. In total, there were 26 different OHCs. These were used for a variety of different health topics including smoking cessation (n=7), cancer (n=6), mental health issues (n=6), diabetes (n=5), multiple sclerosis (n=1), and social innovation in health care (n=1). These OHCs were based in different countries including the United States (n=8), Canada (n=2), Australia (n=1), Germany (n=1), New Zealand (n=1), Norway (n=1), Taiwan (n=1), and the United Kingdom (n=1). The country of origin for 10 OHCs was not reported. The sample of people drawn from each OHC ranged in size from 77 to 49,552 people. Most included between 1000 and 10,000 people; however, one group of 5 OHCs included more than 140,000 people between them. All of the studies were published in 2007 or later, with 12 of the 20 published since 2013.

Table 2 [2,3,12-29] shows a summary of these types of participation. Within Table 2, we have grouped participation styles first into two categories: those based on multiple metrics (multidimensional) and those based on one metric (unidimensional). Each of these is also then divided into up to 3 categories according to the predominant type of metric used to define the participation style: activity-based, network-based, and content-based metrics. Table 3 [30,31] contains a list of the metrics and a description of what they measure.

There were 41 participation styles in the multidimensional category (13 activity based, 11 network based, and 17 content based). In all instances where a unidimensional participation style was identified, the studies divided the users into no more than 3 groups that we have summarized as high, medium, and low engagement. There were 8 different metrics used in the high engagement category (5 activity based, 3 network based), 3 in the medium category (2 activity based, 1 network based), and 4 in the low category (3 activity based, 1 network based).

The results of each subcategory of participation style (content based, network based, and activity based) are described in turn for the 41 multidimensional participation styles. Following this, the results of the unidimensional participation styles are described together for each of the 3 participation styles identified.

**Table 1:** Summary of online health community characteristics.

Online health community name	Year of study	Health condition	Country	Sample size, n
SOL-Cancer Forum	2007 [27]	Cancer	Not reported	84
Cancer Survivors Network	2014 [22,23]	Cancer	United States	27,173
Cancer Compass	2011 [28]	Cancer	United States	7991
WebChoice	2013 [13]	Cancer (breast and prostate)	Norway	103
Breastcancer.org	2014 [29]	Cancer (breast)	United States	49,552
Cancer Compass	2010 [17]	Cancer (melanoma)	United States	851
Five unnamed forums in English and Spanish	2013 [14]	Diabetes	Not reported	>140,000
BlueBoard	2014 [2]	Mental health	Australia	2932
DepressionCenter	2014 [3]	Mental health (depression)	Not reported	5151
PanicCenter	2014 [3]	Mental health (panic disorder)	Not reported	11,372
AlcoholHelpCenter	2014 [3]	Mental health (problem drinking)	Not reported	2597
PTT.CC—Psychosis Support Group	2009 [26]	Mental health (psychosis)	Taiwan	438
SharpTalk	2011 [19]	Mental health (self-harm)	United Kingdom	77
Deutsche Multiple Sklerose Gesellschaft	2014 [20]	Multiple sclerosis	Germany	1169
The Canadian Cancer Society’s Smokers’ Helpline Online	2012 [21]	Smoking	Canada	1670
QuitBlogs	2014 [18]	Smoking	New Zealand	3448
Alt.Support.Stop-Smoking	2014 [25]	Smoking	Not reported	8236
QuitPlan	2008 [12]	Smoking	United States	233
QuitNet	2010 [15]	Smoking	United States	7569
	2013 [16]			Not reported
StopSmokingCenter	2012 [21]	Smoking	United States	1627
	2014 [3]			44,870
#HCSMCA	2013 [24]	Social innovation in health care	Canada	486

**Table 2:** Summary of participation styles including name, metrics, and inclusion criteria.

Name			Metrics	Inclusion criteria
**Multidimensional**				
	**Content based**			
		Influential user [23]	69 activity, network, and content features including influential responding replies	Machine learning classifier (relying initially on expert judgement to identify exemplars)
		Leader [22]	68 activity, network and content features	Machine learning classifier (relying initially on expert judgement to identify exemplars)
		Opinion leader [16]	Word vectors, degree	Latent semantic analysis and high degree
		Information providers [29]	Social support type	High information support
		Community builders [29]	Social support type	High companionship support
		Emotional support providers [29]	Social support type	High emotional support
		Information seekers [29]	Social support type	High information support seeking
		Emotional support seekers [29]	Social support type	High emotional support seeking
		Information enthusiasts [29]	Social support type	High information support seeking, high information support
		All-around contributors [29]	Social support type	No particular metric stands out
		Balanced source user [20]	Source of information	Cited information from a range of sources
		Social media fan [20]	Source of information	High social media
		Organization follower [20]	Source of information	High organizations
		Homepage promoter [20]	Source of information	High static informational websites
		Seeker of health care [20]	Source of information	High health practitioners
		User of uncommon sources [20]	Source of information	High uncommon sources
		Sophisticated contributor [20]	Word count, source of information	High word count, high academic references
	**Network based**			
		Key player [15]	Degree (nonredundant)	Key Player 1.4 software
		Hub [14,17,28]	Out-degree, in-degree	Hyperlink-induced topic search algorithm
		Authority [14,17,28]	Out-degree, in-degree	Hyperlink-induced topic search algorithm
		Facilitator [17,28]	Out-degree, in-degree	Hyperlink-induced topic search algorithm
		Trusted user [14]	Out-degree, in-degree	PageRank algorithm
		Help-seeker [14]	Out-degree, in-degree	Low in-degree, high out-degree (within the scope of the edge between 2 users)
		Star [27]	Out-degree, in-degree	Top ranked individual (outlier)
		Prime givers [14,27]	Out-degree, in-degree	Very high out-degree, high in-degree
		Serious members [27]	Out-degree, in-degree	Moderate out-degree, moderate in-degree
		Moderate users [27]	Out-degree, in-degree	Low out-degree, low in-degree
		Takers [27]	Out-degree, in-degree	No out-degree, low in-degree
	**Activity based**			
		Caretaker [19]	Time logged in, episodes, reading, posting, thread initiation	High time logged in, low episodes, high reading, low posting, low thread initiation
		Here for you [19]	Thread initiation, posting, forum	Low thread initiation, high posting in support forum
		Butterfly [19]	Time logged in, episodes, posting, forum	High time logged in, high episodes, high posting in support forum
		Crisis-oriented individual [19]	Posting, forum	High posting in support forum
		Discussant [19,20]	Thread initiation, posting, forum	High thread initiation, high posting in discussion forum
		Average user [20]	Thread initiation, posting, forum, topic, days active, word count, source of information	No particular metric stands out
		Highly active relational poster [20]	Posts per day, thread participation, thread initiation	High posts per day, high thread participation, low thread initiation
		Topic-focused responder [20]	Thread initiation, posting, topic, days active	Low thread initiation, low posts per day, high fraction of topic-related posts, low days active
		Topic-spammer [20]	Posting, days active, word count, topic, source of information	Low days active, high posting, low word count, high fraction of topic-related posts, low references
		Long-term high-activity users [25]	Days active, posting	High days active, high posting
		Short-term high-activity users [25]	Days active, posting	Low days active, high posting
		Short-term low-activity users [25]	Days active, posting	Low days active, low posting
		Long-term low-activity users [25]	Days active, posting	High days active, low posting
**Unidimensional**				
	**Activity based**			
		High-engaged user	Posting	>2 posts [12]; top 1% of users [2,3]; top 10 users [24]; >180 posts [18]; top 100 users [21]
			Reading	>5 posts [12]
			Time logged in	Top 33.3% of users [13]
			Thread initiation	Top 100 users [21]
			Thread participation	Top 100 users [21]
			Network based	
			Friendship	Mutual friend nomination between 2 users and >4 interactions between them [15]
			In-degree	Top 10 users [24]; high in-degree [26]
			Out-degree	Top 10 users [24]; high out-degree [26]
		Moderate-engaged user	Posting	2-10 percentile (9%) of users [2,3]
			Time logged in	Middle 33.3% of users [13]
			Network based	
			Friendship	Friend nomination of another user and >0 interactions with them [15]
		Low-engaged user	Posting	1-2 post [12,18]; bottom 90% of users [2,3]
			Reading	1-5 posts [12]
			Time logged in	Bottom 33.3% of registered users [13]
			Network based	
			Friendship	Any interactions with another user [15]

**Table 3:** A description of the mtrics used to classify participation styles.

Metric		Description
Activity-based metrics: measure the individual actions taken by users in an OHC		
	Posting	Number of posts a person has made in the OHC
	Time logged in	Amount of time a person has spent accessing the OHC
	Reading	Number of posts that a person has read
	Thread initiation	Number of times a person has created a thread
	Episodes	Number of times a person has accessed the OHC
	Days active	Number of days between a person’s first and last post
	Forum	Number of posts a person has made in a particular subforum of the OHC, eg, support or discussion
	Thread participation	Number of different threads a person has posted in
Network-based metrics: measure the relationship and interactions between users		
	Degree (in/out)	The number of people a person has communicated with. Where it is possible to tell who the source of the communication was and to whom it was directed, the number of people a person has made outgoing communication with is called the “out-degree” and the number of people that a person has received communication from is called the “in-degree.” When it is not possible to tell the direction, the communication is counted for both people as a measure of degree. Degree is considered to be a measure of a user’s centrality in a network [30,31].
	Friendship	The extent to which a person is connected with at least one other person in the OHC as defined by 3 thresholds: Low—any interactions with another user; Moderate—friend nomination of another user and >0 interactions with them; and High—mutual friend nomination between 2 users and >4 interactions between them.
Content-based metrics: measure the nature of the content within posts		
	Word vectors	A representation of the proportion of words in a message that fit a certain topic.
	Influential Responding Replies	Number of posts a person has made that have influenced the sentiment of the thread initiator
	Social support type	Number of posts a person has made that either provide or seek information support, emotional support, or companionship
	Topic	Number of posts a person has made which included subject matter on a specific topic
	Source of information	Number of citations a person has made from a particular source
	Word count	Number of words in a post

### Multidimensional

#### Content-Based

##### Leaders and Influential Users

Zhao et al [23] created a machine learning classifier with 69 metrics that was used to identify influential users in an OHC. These users were regarded as leaders who could influence the emotional sentiment of other users. This study built on previous research by Zhao et al [22], which used 68 metrics such as number of posts, in-degree, and days active in a classifier to first identify leaders in the OHC. Zhao et al [23] then created a metric called “influential responding replies (IRRs).” This was the number of times a person was able to affect the sentiment of another person when responding to their initial post. It was found that this metric alone outperformed the classifier with 68 metrics, and together they created the best performing classifier. In order to train this IRR-enhanced classifier, it was necessary to have a list of users who were deemed to be influential users by moderators of the OHC. There were 41 users in this list. In total, the moderators identified 126 influential users. A list of the top 50, 100, and 150 influential users identified by the classifier was made up with 90%, 77.7%, and 68.7% users from the moderator list of 126 respectively. The highest percentage possible in the 150 influential user condition was 84.0% (126/150).

##### Opinion Leaders

Myeni et al [16] used latent semantic analysis to identify users who were involved in discussion about particular concepts such as personal experiences, advice, or adherence to interventions. Users whose mean word vector scores for a concept were one standard deviation above the sample mean were grouped together in a social network. Within each theme-based social network, Opinion Leaders were identified as people who had the highest degree. These people were considered to be influential in their specific domain and may be particularly useful to identify when delivering relevant targeted interventions. Subsequent research has shown that exposure to users who were abstinent from smoking in the theme-based networks of “social support” and “traditions” were more likely to be abstinent themselves [32].

##### Information Providers, Emotional Support Providers, Information Support Seekers, Emotional Support Seekers, Community Builders, Information Enthusiasts, and All-Around Contributors

Wang et al [29] created a machine learning classifier to determine which posts in a cancer OHC with more than 2.8 million posts contained each of the following types of content: providing informational support, providing emotional support, seeking informational support, and seeking emotional support and companionship. The authors then used a k-means clustering algorithm [33] to categorize all users based on the proportion of posts they made with each type of content. This produced 7 types of users. Five were typified by writing a high proportion of posts that predominantly contained one type of each of the above 5 content types. The remaining two, information enthusiasts and all-around contributors, were typified by having equally high proportions of posts seeking and providing informational support, and having equal amounts of all types of content, respectively. The all-around contributor was the most common type of user of all 7 (making up 32% of all users). Community builders were among the least common (8%) but were responsible for writing the most posts on average along with all-around contributors. Those who primarily engaged in informational and emotional support types posted less and did not remain in the forum as long as community builders and all-around contributors.

##### Balanced Source User, Social Media Fan, Organization Follower, Homepage Promoter, Seeker of Health Care, and User of Uncommon Sources

Sudau et al [20] observed that people tend to favor different sources of information to support the points that they make in posts. A number of participation styles represent this bias. In order to determine these participation styles, Sudau et al used a k-means clustering algorithm [33] to form 6 groups of similar users based on the frequency of different hyperlinks they used from 8 domain classes. The groups were labeled according to what Sudau et al thought best described their referencing tendencies.

##### Sophisticated Contributor

A sophisticated contributor is a user whose posts are longer than those of the average user participation style and contain more references. In contrast to the activities of most users, these references are more often to scientific publications than to social media sources. Sudau et al [20] identified this participation style in 4 of 171 users. Sophisticated contributor posts were three times as long and contained five times as many references as posts by Average Users.

#### Network Based

##### Key Players

Cobb et al [15] sought to identify a set of users who were maximally connected to other users throughout the social network of the OHC. A set of key players is a small group of a specified number of users who are connected with as many other people in the network as possible, for example, through private message, posting, or friendship. Cobb et al used Key Player 1.4 software [34] to determine the reach of a set of 50 key players. These 50 key players were connected to 64% of other users in the network. Note that these are not necessarily the 50 most connected individuals in the OHC; that is, they are not the top 50 users ranked by degree. Rather, the algorithm considers redundancy. If introducing a new key player to the set does not increase the set’s overall reach, that player is not added. The optimum key player set of 50 users may not necessarily contain all the users in the 49 set nor will either necessarily contain the user who, as an individual, is the most connected person in the network. The intention of the algorithm is to enable maximum access to the whole network through minimal nodes. This, for example, enables maximum efficiency in dissemination of information.

##### Hubs, Authorities, and Facilitators

Hubs and authorities are concepts borrowed from the computer science literature on the Web. Hubs and authorities are identified using the hyperlink-induced topic search (HITS) algorithm [35]. In this algorithm, every website receives both a hub and an authority score. High-scoring authorities are websites that are linked to high-scoring hubs. High-scoring hubs are websites that link to high-scoring authorities. Websites with high authority scores tend to those that provide good information on a specific topic. Hubs direct people to these various authorities. The algorithm can be applied to any network consisting of nodes and links between them by analyzing the pattern of out-degree and in-degree across the network. Accordingly, both Chomutare et al [14] and Durant et al [17,28] have used the HITS algorithm to identify people in OHCs as authorities and hubs. The 3 papers have posited that those identified as hubs are people who disseminate information by promoting discussion. They have a relatively high out-degree in the network compared with their in-degree. They are important for sustaining the activity levels of the community. Authorities are people whose opinion is highly respected in the community. They have a relatively high in-degree. A third participation style—a facilitator—was also proposed by Durant et al [17]. A facilitator is a person who is ranked similarly highly as a hub and as an authority. They are considered to be more effective for sustaining communication in the OHC than those who are hubs or authorities alone. Durant et al [28] sought to track the presence of facilitators over time by segmenting and analyzing the network each year over an 8-year period and found that the top 5% were rarely the same individuals in consecutive years.

##### Trusted Users

Similar to the HITS algorithm, the PageRank algorithm [36] is another method originating in the computer science literature on the Web. Rather than identifying 2 types of users, the PageRank algorithm identifies one type. The score given to each node in the network by the PageRank algorithm is the probability of arriving at that node given a random walk around the network via the links between them. This means that nodes that are linked to more often have higher probabilities of being landed on, and nodes that are linked to more often by other high-scoring nodes have even higher scores. If it is assumed that a directional link between 2 nodes is a vote of support to the other, this algorithm identifies trusted users. This algorithm was the basis for Google search. Chomutare et al [14] have applied it to an OHC and have made the same assumption. They found that 6 out of 10 of the highest ranked users by in-degree were also in the top 10 identified by the PageRank algorithm.

##### Help-Seekers

In a relationship between 2 people where one communicates with the other much more often, the person who instigates more communication (higher out-degree than in-degree) is labeled a Help-Seeker. Chomutare et al [14] suggested that this pattern of metrics might reflect a person who is struggling with their health issue. However, the authors noted that the nature of the help-seeking is not exactly clear as the user may either be strongly motivated to engage in self-care or they may be a particularly needy user, and neither can be concluded without content analysis. The authors originally suggested the label “needy user” for this participation style, but we have renamed it “help-seeker” given the ambiguity and lack of clarity around the concept of needy in this context.

##### Star, Prime Givers, Serious Members, Moderate Users, and Takers

The earliest recorded participation styles were identified by Bambina [27] who compared the in-degree and out-degree of users and grouped them around a pattern in the results that was related to engagement. Bambina first noted one outlier: a person who had both the highest in-degree and out-degree. Bambina referred to this person as the “star.” This person provided the most support to others including notably many new individuals with whom many others did not communicate. Bambina noted that the next most engaged people by both in-degree and out-degree all tended to provide more support than they received, that is, have higher out-degree than in-degree. These were named “prime givers” (n=6). Chomutare et al [14] observed the same pattern in a social network analysis that they conducted, but they did not report whether it was associated with providing support. Bambina also noted 2 groups who had relatively similar in-degree and out-degree within each group. These were the designated “serious members” (10) and “moderate users” (n=15). Last was a group labeled the “takers” who never provided support but who initiated a conversation and received support from others (n=52).

#### Activity-Based

##### Caretakers

Jones et al [19] identified one user in a sample of 77 people as having a participation style called the “caretaker.” They identified this person, as they did for all participation styles, through visual inspection of scatterplots of various metrics. The OHC was a support group for young people who self-harm. Given the large amount of time the person spent logged in, they actively participated very little. The times they did post were largely in response to other users rather than initiating their own threads. Jones et al concluded that this person might be watching over the whole forum and looking out for others in need. This person undertook the caretaker role despite the OHC being a moderated forum.

##### Here for You

One user in a sample of 77 people was considered to take the “here for you” participation style by Jones et al [19]. Like the caretaker, they did not create many threads of their own. However, in contrast to the latter, they did post large amounts of comments in response to other people who needed support.

##### Butterfly

Another user in the Jones et al [19] sample was classified as being characterized by a butterfly participation style. This person logged in many more times than anyone else. They spent short amounts of time checking out a few pages and then logged out again. They posted mostly in the support forum (as opposed to the discussion forum or off-topic forum). Like the crisis-oriented individuals in the following section, they were considered by the moderators to be in crisis and needing support as opposed to providing it.

##### Crisis-Oriented Individuals

Six users of the Jones et al [19] sample were classified as crisis-oriented in their participation style. These people posted in large numbers in the support forum. It is not possible to confirm from the objective metrics alone whether such people were in crisis or providing support; however, it was confirmed by the moderators of the forum that all 6 were indeed in crisis. These users did not visit the OHC as frequently as the user with the butterfly participation style.

##### Discussants

A discussant is a user who is mainly focused on discussion about health-related topics as opposed to providing or receiving support. They initiate a high number of threads in the discussion section of the OHC and participate actively in them. This participation style was identified by both Jones et al [19] and Sudau et al [20].

##### Average Users

A user type that is not distinctly based on any metric, the average user category was identified by the application of a second k-means clustering algorithm conducted by Sudau et al [20]. This analysis was designed to form 6 groups of similar users based on 9 metrics that measured their active participation in the community. Sudau et al labeled the groups according to their distinguishing features. Average users were a group that were thought not to exhibit any distinguishing features. This group constituted 63% of the people included in the analysis.

##### Highly Active Relational Posters

These are the most active users of an OHC by post frequency. Sudau et al [20] noted these users maintain “small talk,” which may be good for community building. They participate in many different threads but do not initiate many themselves.

##### Topic-Focused Responders

A user whose activity is concentrated on a specific topic, the topic-focused responder is distinct from a discussant in that they do not post as much and do not initiate as many threads. Sudau et al [20] included only people who had made at least five posts on a certain topic in their analysis. Topic-focused responders met this criterion but they did not have many other posts. They tended to focus mainly on responding to others who had initiated the topic. Sudau et al suggested this style may be similar to the here for you participation style identified by Jones et al [19], but we have separated them because of the distinction between discussion and support.

##### Topic-Spammers

This is a user who is active for a very short period, that is, only a few days. In that time, they contribute a high number of posts on a specific topic in the discussion forum. However, these are not particularly sophisticated posts, rather they are short and lack references. This participation style was identified by Sudau et al [20].

##### Short-Term and Long-Term, High-Activity and Low-Activity Users

Stearns et al [25] noted that the bulk of users in a smoking cessation OHC are made up of short-term users (active for approximately less than 1 week), who, regardless of whether they have high or low activity, tend to be involved in the OHC for personal gain. Long-term users with low activity are noted to have smaller social circles and a stronger interest in particular topics. Stearns et al state that long-term high-activity users are most like Young’s [5] “core members” who are vital to the sustainability of the OHC.

### Unidimensional

All but one of the studies [24] that made unidimensional classifications did so for the purpose of determining if the type and level of engagement a person showed was predicted by demographic factors and whether high engagement predicted specific health outcomes. Some studies made statements about the nature of participation of users in the OHC. Given that the purpose of this review was to investigate the nature of participation, we focus on reporting these findings in the following sections considering first high-engaged users, followed by moderate- and low-engaged users.

#### High-Engaged Users

All 8 studies that made a unidimensional categorization [2,3,12,13,15,18,21,24] classified users into a participation style that we call high-engaged users. There were 8 different metrics used across these studies that all indicate a different type of high engagement. These included posting frequency, thread initiation, thread participation, level of in-degree/out-degree, reading of posts, time logged in, and friendship (see Table 3 for definitions).

Frequency of posting was the most commonly used metric used by 6 of the 8 studies [2,3,12,18,21,24]. It was used to classify users in a total of 9 OHCs, with 4 of those being for smoking cessation, 4 for mental health issues, and 1 for social innovation in health care. Users who were highly engaged according to posting frequency were regarded by all but one of the studies [12] as being valuable to the OHC because they sustained activity levels and in doing so facilitated the engagement of others. Four of the 6 studies referred to these people as either “superusers” [2,3,21] or “community leaders” [24]. This regard spanned across all the types of OHCs mentioned earlier.

Thread initiation and thread participation (together with posting frequency) were used by one study [21] to classify the top 100 ranked users, denoting them “superusers.” The moderators of the OHC were asked to identify leaders within it. The authors noted that although most studies have previously identified leaders in an OHC using posting frequency alone, the moderators thought it was necessary to also include users who start many conversations and who participate in many different conversations in their definition of a “superuser.”

In-degree and out-degree were employed by 2 studies to classify users as highly engaged [24,26]. The authors of one study [24] regarded users with high in-degree (top 10) as authorities on topics, similar to the hubs and authorities discussed earlier. This study was conducted on an OHC that existed within Twitter. It was noted that those people with the highest in-degree were also people who had the highest number of followers on Twitter in general. Users with high in-degree were considered to be valuable for engaging other less active users in discussion. It was noted that the 6 users were both top 10 ranked users by in-degree and out-degree. These 6 people were thought to be communicating on topics that resonated with the community and were considered to be “community leaders.” In a study of a mental health OHC for psychosis, Chang et al [26] referred to users with either a high in-degree or out-degree as “stars” after Bambina’s [27] single outlying user.

Other metrics employed to classify users as highly engaged included reading [12], time logged in [13], and friendship [15].

#### Moderate-Engaged Users

Four studies classified users as moderately engaged based on 3 different metrics. Two were based on posting frequency [2,3], and one each on time logged in [13] and friendship [15].

#### Low-Engaged Users

Six studies classified users as low engaged based on 4 different metrics. Four were based on posting frequency [2,3,12,18], and one each on reading [12], time logged in [13], and friendship [15].

## Discussion

### Principal Findings

This systematic review synthesized findings from studies that investigated the nature of participation in an OHC by categorizing users based on metrics of participation. The aim of this review was to identify the different ways in which users participate and contribute to OHCs, although we acknowledge that the resultant list of participation styles may not provide a comprehensive account of all possible styles. Our objective was to determine whether any patterns were apparent in the types of participation styles that were identified across and within different health conditions. With the exception of an overlap in engagement measured by posting frequency (which has been discussed elsewhere [3]), there was little overlap in participation styles identified across OHCs for different health conditions or within OHCs for specific health conditions. Consequently, it is not possible for this study to address this objective. This area of research is in its infancy, with most of the studies included in this review being published in the last 2 years. Despite this shortcoming, the current review delivers a nomenclature for OHC participation styles and metrics that will provide a basis for future comparative research in the area. To inform future research, we discuss in the following section some methodological considerations for studies seeking to replicate or expand on the methods identified by this review.

### Methodological Considerations

#### Posting Frequency

It was common for studies to use posting frequency as the sole means of classifying highly engaged users in an OHC. It was also common among these studies for researchers to regard these users as being particularly valuable to the OHC. However, it is not possible to know from post frequency alone in what way a person is contributing to an OHC. They might be contributing trivial or critical messages or their post might in other ways fail to support others. The rationale for the inference that high engagement is synonymous with high value may relate to another commonality across papers. The authors in question were also community managers of the OHCs that they were studying; therefore, they may have based their conclusions on reading content posted by these users. However, content analysis research is required to investigate whether posting frequency is a valid means of identifying generically valuable users.

#### Machine Learning

Zhao et al [22,23] used a complex method of identifying the participation styles of leaders and influential users that may be subject to issues with generalizability. Ideally, the classifier would be transferable across OHCs. However, there is currently no evidence to support such transferability. Indeed by using 69 metrics in their machine learning classifier, they may have created a model that is overfitted to the data of the OHC from which it came and it may not work well at identifying leaders or influential users in other OHCs, even of the same health condition. Furthermore, an essential prerequisite for the development of the method was identifying a priori, using subjective judgments, a sample of leaders and influential users for use in the learning classifier trial. Thus, if Zhao’s classifier is not generalizable, research to identify a new model requires expertise, or access to expertise, in identifying leaders and influential users through qualitative methods in addition to advanced understanding of machine learning methods. Despite these challenges, research in this area offers promise, particularly as influential users most closely resemble those vital users whom Young [5] described as core members. For those who are not inclined to build their own classifier, it is noteworthy that one particularly useful and generalizable aspect of the method for determining influential users was the discovery of the metric influential responding replies, which is defined as the number of posts a person has made that have influenced the sentiment of the thread initiator. Zhao reported that this metric was a better predictor of influential user status than the other 68 metrics combined. IRRs are determined by analyzing the degree of positive and/or negative sentiment expressed in the text. There are many existing programs that can conduct this kind of sentiment analysis, such as Linguistic Inquiry and Word Count [37]. However, note that it is important to test the validity of these programs in any new dataset by comparing human and computer ratings. As Zhao points out, the word “positive” in the context of a cancer diagnosis can be a negative concept. Applying a standard sentiment analysis program in this context would yield invalid results.

Wang et al [29] also used a machine learning classifier; however, their method may be more reliably replicated without expert knowledge. The classifier was designed to detect the presence or absence of certain types of social support in posts. They used 5 human coders to classify a sample of posts that could be used for training the classifier. These people were not domain experts. Similar research has involved contracting online Amazon Mechanical Turk workers to code the presence of social support in posts for the same purpose [38]. These people also did not have prior experience in this area.

#### Centrality Algorithms

Similar to IRR, some participation styles described users who were useful in a particular way that would be potentially identifiable in any OHC, or for that matter, any social network. These were based on algorithms that used measures of centrality such as in-degree and out-degree. This includes authorities, hubs, facilitators, and trusted users. While these categories are quite useful, it should be noted that these algorithms are calculated in such a way that they introduce bias based on time elapsed such that users who participate earlier in the OHC receive higher scores [39]. There are methods to adjust for this [40].

#### K-means Clustering and Multivariate Outliers

Other more specific participation styles described users who have particular characteristics and may be found only in a subset of OHCs. This included, for example, the caretaker or the topic-spammer. The techniques used to identify these participation styles, k-means clustering algorithms and multivariate outliers, may not necessarily identify the same participation styles in other OHCs. However, they may be useful for identifying other particular or unique ways of participating in OHCs.

### Limitations and Future Research

The scope of this study is quite broad. We included all studies that categorized a type of participation in an OHC despite the possibility that the culture and nature of participation in populations with different health conditions and with or without moderators could differ markedly. There was little overlap in the use of categorizations to define particular participation styles either in OHCs broadly or within specific health conditions. Thus, it is not possible to draw many specific conclusions at this early stage. A possible limitation and reason for this is that we may not have included all relevant studies, as our search terms may not have encompassed all the different terms used to describe participation styles at this early stage of research. Nevertheless, by synthesizing the findings of the included studies, this review provides a basis for future research to investigate the validity of styles identified to date by attempting to replicate findings for specific OHCs and exploring their validity across different OHCs. Future research should also investigate new participation styles not documented in this review.

### Conclusion

Our systematic review identified a range of participation styles. Some of them may be generalizable to other OHCs. Others were more specific to particular OHCs but were identified by methods that could be used elsewhere. The findings of this review are intended to support the work of community managers in building community, organizations seeking to design targeted interventions and disseminate information through certain types of people in OHCs, and researchers seeking to understand the nature of peer support. We anticipate that this review will be useful for these groups in conducting investigations to determine the presence of participation styles that may be relevant to their work. However, it is too early to draw any conclusions about which OHCs would be most likely to contain users who have specific participation styles.

## Appendix

Multimedia Appendix 1 OHC concept search terms.

## Abbreviations

HITS: hyperlink-induced topic search
IRRs: influential responding replies
OHC: online health community
