# Supplementary material for: From Help-Seekers to Influential Users: A Systematic Review of Participation Styles in Online Health Communities
Source: J Med Internet Res. 2015 Dec 1;17(12):e271. doi: 10.2196/jmir.4705 (PMC4704975; doi:10.2196/jmir.4705)
Supplement: Multimedia Appendix 1 [file jmir_v17i12e271_app1.pdf]

Database/Notes	Terms
**PubMed**	
#1	Search "computer communication network"[Title/Abstract] OR "computer-network"[Title/Abstract] OR "computer-based"[Title/Abstract] OR "computer-generated environment"[Title/Abstract] OR cyber[Title/Abstract] OR cyberspace[Title/Abstract] OR "data highway"[Title/Abstract] OR electronic[Title/Abstract] OR "electronic mail"[Title/Abstract] OR email[Title/Abstract] OR "e-mail"[Title/Abstract] OR "information highway"[Title/Abstract] OR "information superhighway"[Title/Abstract] OR "information super highway"[Title/Abstract] OR internet[Title/Abstract] OR "internet-based"[Title/Abstract] OR net[Title/Abstract] OR online[Title/Abstract] OR virtual[Title/Abstract] OR web[Title/Abstract] OR "web-based"[Title/Abstract] OR webbased[Title/Abstract] OR "web page"[Title/Abstract] OR webpage[Title/Abstract] OR "web site"[Title/Abstract] OR website[Title/Abstract] OR "world wide web"[Title/Abstract] OR www[Title/Abstract]
#2	Search “health community”[Title/Abstract] OR “health communities"[Title/Abstract] OR "discussion group"[Title/Abstract] OR forum[Title/Abstract] OR "interpersonal communication"[Title/Abstract] OR "interpersonal interaction"[Title/Abstract] OR "multi-user"[Title/Abstract] OR network[Title/Abstract] OR "peer support"[Title/Abstract] OR "peer-support"[Title/Abstract] OR "peer to peer"[Title/Abstract] OR p2p[Title/Abstract] OR selfhelp[Title/Abstract] OR "self-help"[Title/Abstract] OR "social network"[Title/Abstract] OR "social support"[Title/Abstract] OR "support group"[Title/Abstract] OR "support network"[Title/Abstract] OR "support system"[Title/Abstract]
#3	Search (#1 AND #2)
#4	Search ("internet community"[Title/Abstract] OR "internet-based community"[Title/Abstract] OR "world wide web community"[Title/Abstract] OR "e-mail community"[Title/Abstract] OR "email community"[Title/Abstract] OR "electronic mail community"[Title/Abstract] OR "web community"[Title/Abstract] OR "web page community"[Title/Abstract] OR "webpage community"[Title/Abstract] OR "web site community"[Title/Abstract] OR "website community"[Title/Abstract] OR "web-based community"[Title/Abstract] OR "webbased community"[Title/Abstract] OR "www community"[Title/Abstract] OR "cyber community"[Title/Abstract] OR "virtual community"[Title/Abstract] OR "online community"[Title/Abstract] OR "e-community"[Title/Abstract] OR "electronic

	community"[Title/Abstract])
#5	Search ("internet society"[Title/Abstract] OR "internet-based society"[Title/Abstract] OR "world wide web society"[Title/Abstract] OR "e-mail society"[Title/Abstract] OR "email society"[Title/Abstract] OR "electronic mail society"[Title/Abstract] OR "web society"[Title/Abstract] OR "web page society"[Title/Abstract] OR "webpage society"[Title/Abstract] OR "web site society"[Title/Abstract] OR "website society"[Title/Abstract] OR "web-based society"[Title/Abstract] OR "webbased society"[Title/Abstract] OR "www society"[Title/Abstract] OR "cyber society"[Title/Abstract] OR "virtual society"[Title/Abstract] OR "online society"[Title/Abstract] OR “e-society"[Title/Abstract] OR "electronic society"[Title/Abstract])
#6	Search ("internet environment"[Title/Abstract] OR "internet-based environment"[Title/Abstract] OR "world wide web environment"[Title/Abstract] OR "e-mail environment"[Title/Abstract] OR "email environment"[Title/Abstract] OR "electronic mail environment"[Title/Abstract] OR "web environment"[Title/Abstract] OR "web page environment"[Title/Abstract] OR "webpage environment"[Title/Abstract] OR "web site environment"[Title/Abstract] OR "website environment"[Title/Abstract] OR "web-based environment"[Title/Abstract] OR "webbased environment"[Title/Abstract] OR "www environment"[Title/Abstract] OR "cyber environment"[Title/Abstract] OR "virtual environment"[Title/Abstract] OR "online environment"[Title/Abstract] OR "e-environment"[Title/Abstract] OR "electronic environment"[Title/Abstract])
#7	Search ("bulletin board system"[Title/Abstract] OR "chat group"[Title/Abstract] OR chatgroup[Title/Abstract] OR "chat room"[Title/Abstract] OR chatroom[Title/Abstract] OR "chat technology"[Title/Abstract] OR "discussion list"[Title/Abstract] OR "ebulletin board"[Title/Abstract] OR "e-bulletin board"[Title/Abstract] OR "electronic bulletin board"[Title/Abstract] OR "instant messaging"[Title/Abstract] OR "internet relay"[Title/Abstract] OR irc[Title/Abstract] OR "list serv"[Title/Abstract] OR listserv[Title/Abstract] OR "mail box"[Title/Abstract] OR mailbox[Title/Abstract] OR "mailing list"[Title/Abstract] OR "message board"[Title/Abstract] OR messageboard[Title/Abstract] OR "news group"[Title/Abstract] OR newsgroup[Title/Abstract] OR usenet[Title/Abstract])
#8	Search ("computer-based group"[Title/Abstract] OR "computer-based support"[Title/Abstract] OR "computer-based interaction"[Title/Abstract] OR "computer-based intervention"[Title/Abstract] OR "cyber

	group"[Title/Abstract] OR "cyber support"[Title/Abstract] OR "cyber interaction"[Title/Abstract] OR "cyber intervention"[Title/Abstract] OR "electronic support"[Title/Abstract] OR "electronic group"[Title/Abstract] OR "electronic interaction"[Title/Abstract] OR "electronic intervention"[Title/Abstract] OR "e-support"[Title/Abstract] OR "e-group"[Title/Abstract] OR "e-interaction"[Title/Abstract] OR "e-intervention"[Title/Abstract] OR "internet group"[Title/Abstract] OR "internet support"[Title/Abstract] OR "internet-based group"[Title/Abstract] OR "internet-based support"[Title/Abstract] OR "online group"[Title/Abstract] OR "online support"[Title/Abstract] OR "online network"[Title/Abstract] OR "virtual support"[Title/Abstract] OR "virtual group"[Title/Abstract] OR "virtual interaction"[Title/Abstract] OR "virtual intervention"[Title/Abstract] OR "web group"[Title/Abstract] OR "web support"[Title/Abstract] OR "web interaction"[Title/Abstract] OR "web intervention"[Title/Abstract] OR "web-based group"[Title/Abstract] OR "web-based support"[Title/Abstract] OR "web-based interaction"[Title/Abstract] OR "web-based intervention"[Title/Abstract] OR "web-based program"[Title/Abstract] OR "online interaction"[Title/Abstract] OR "online intervention"[Title/Abstract])
#9	Search (#3 OR #4 OR #5 OR #6 OR #7 OR #8)

PsycInfo

#1	(computer communication network or computer-network or computer-based or computer-generated environment or cyber or cyberspace or data highway or electronic or electronic mail or email or e-mail or information highway or information super highway or information superhighway or internet or internet-based or net or online or virtual or web or web-based or webbased or web page or webpage or web site or website or world wide web or www).ab,ti.
#2	(health community or health communities or discussion group or forum or interpersonal communication or interpersonal interaction or multi-user or network or peer-support or peer support or peer to peer or p2p or selfhelp or self-help or social network or social support or support network or support group or support system).ab,ti.
#3	1 and 2
#4	(internet community or internet-based community or world wide web community or e-mail community or email community or electronic mail community or web community or web-based community or webbased community or web page community or webpage community or website community or web site community or www community or cyber community or

	virtual community or online community or e-community or electronic community).ab,ti.
#5	(internet society or internet-based society or world wide web society or e-mail society or email society or electronic mail society or web society or web page society or webpage society or web site society or website society or web-based society or webbased society or www society or cyber society or virtual society or online society or e-society or electronic society).ab,ti.
#6	(internet environment or internet-based environment or world wide web environment or e-mail environment or email environment or electronic mail environment or web environment or web page environment or webpage environment or web site environment or website environment or web-based environment or webbased environment or www environment or cyber environment or virtual environment or online environment or e-environment or electronic environment).ab,ti.
#7	(bulletin board system or chat group or chatgroup or chat room or chatroom or chat technology or discussion list or ebulletin board or e-bulletin board or electronic bulletin board or instant messaging or internet relay or irc or list serv or listserv or mail box or mailbox or mailing list or message board or messageboard or news group or newsgroup or usenet).ab,ti.
#8	(computer-based group or computer-based support or computer-based interaction or computer-based intervention or cyber group or cyber support or cyber interaction or cyber intervention or electronic support or electronic group or electronic interaction or electronic intervention or e-support or e-group or e-interaction or e-intervention or internet group or internet support or internet-based group or internet-based support or online group or online support or online interaction or online intervention or online network or virtual support or virtual group or virtual interaction or virtual intervention or web group or web support or web interaction or web intervention or web-based group or web-based support or web-based interaction or web-based intervention or web-based program).ab,ti.
#9	3 or 4 or 5 or 6 or 7 or 8

Cochrane

#1	"computer communication network" or "computer-network" or "computer-based" or "computer-generated environment" or cyber or cyberspace or "data highway" or electronic or "electronic mail" or email or "e-mail" or "information highway" or "information superhighway" or "information super highway" or

internet or "internet-based" or net or online or virtual or

web or "web-based" or webbased or "web page" or webpage or "web site" or website or "world wide web" or www

***Search: in title abstract keywords* in Cochrane Reviews (Reviews only) and Trials (Word variations have been searched)**

#2 “health community” or “health communities” or "discussion group" or forum or "interpersonal communication" or "interpersonal interaction" or "multi-user" or network or "peer support" or "peer-support" or "peer to peer" or p2p or selfhelp or "self-help" or "social network" or "social support" or "support group" or "support network" or "support system"

***Search: in title abstract keywords* in Cochrane Reviews (Reviews only) and Trials (Word variations have been searched)**

#3 #1 AND #2

#4 "internet community" OR "internet-based community" OR "world wide web community" OR

"e-mail community" OR "email community" OR "electronic mail community" OR "web community" OR "web page community" OR "webpage community" OR "web site community" OR "website community" OR "web-based community" OR "webbased community" OR "www community" OR "cyber community" OR "virtual community" OR "online community" OR "e-community" OR "electronic community"

***Search: in title abstract keywords* in Cochrane Reviews (Reviews only) and Trials (Word variations have been searched)**

#5 "internet society" OR "internet-based society" OR "world wide web society" OR "e-mail society" OR "email society" OR "electronic mail society" OR "web society" OR "web page society" OR "webpage society" OR "web site society" OR "website society" OR "web-based society" OR "webbased society" OR "www society" OR "cyber society" OR "virtual society" OR "online society" OR “e-society" OR "electronic society"

***Search: in title abstract keywords* in Cochrane Reviews (Reviews only) and Trials (Word variations have been searched)**

#6	"internet environment" OR "internet-based environment" OR "world wide web environment" OR<br><br>"e-mail environment" OR "email environment" OR "electronic mail environment" OR "web environment" OR "web page environment" OR "webpage environment" OR "web site environment" OR "website environment" OR "web-based environment" OR "webbased environment" OR "www environment" OR "cyber environment" OR "virtual environment" OR "online environment" OR "e-environment" OR "electronic environment"<br><br>***Search: in title abstract keywords* in Cochrane Reviews (Reviews only) and Trials (Word variations have been searched)**
#7	"bulletin board system" OR "chat group" OR chatgroup OR "chat room" OR chatroom OR "chat technology" OR "discussion list" OR "ebulletin board" OR "e-bulletin board" OR "electronic bulletin board" OR "instant messaging" OR "internet relay" OR irc OR "list serv" OR listserv OR "mail box" OR mailbox OR "mailing list" OR "message board" OR messageboard OR "news group" OR newsgroup OR usenet<br><br>***Search: in title abstract keywords* in Cochrane Reviews (Reviews only) and Trials (Word variations have been searched)**
#8	"computer-based group" OR "computer-based support" OR "computer-based interaction" OR<br><br>"computer-based intervention" OR "cyber group" OR "cyber support" OR "cyber interaction" OR<br><br>"cyber intervention" OR "electronic support" OR "electronic group" OR "electronic interaction" OR "electronic intervention" OR "e-support" OR "e-group" OR "e-interaction" OR "e-intervention" OR "internet group" OR "internet support" OR "internet-based group" OR "internet-based support" OR "online group" OR "online support" OR "online network" OR "virtual support" OR "virtual group" OR "virtual interaction" OR "virtual intervention" OR "web group" OR "web support" OR "web interaction" OR "web intervention" OR "web-based group" OR "web-based support" OR "web-based interaction" OR "web-based intervention" OR "web-based program" OR "online interaction" OR "online intervention"<br><br>***Search: in title abstract keywords* in Cochrane Reviews (Reviews only) and Trials (Word variations have been searched)**
#9	#3 OR #4 OR #5 OR #6 OR #7 OR #8
